# Miscellaneous

**Published:** 1890-08

**Authors:** 


### MISCELLANEOUS.

### THE MYSTERY OF THE MAGNET.

With all the paraphernalia of the modern physical and electrical laboratory, the instruments of precision that will handle a millionth of an inch as readily as a laborer his pick and shovel ; with all the evolution going on through generations of scientists and the almost incessant wrestling of secrets from the bosom of nature, we doubt if science is any closer to the isolation or intenuation of the microbe of the magnet. It is absurb to suppose that a primary energy is impressed on a piece of hardened steel once for all. The transfer of that energy into actual work would destroy the magnetic power, yet such destruction not only does not take place, but the very exercise of the power strengthens the magnet. A horse-shoe of steel may be magnetized in ten seconds by the current of a few amperes from a battery, a ridiculously small amount of energy all told, and such magnet can lift many pounds of iron in contact. But without contact it may lift and hold a pound of iron easily. It will hold that pound for an eternity, and every second of that time without end the magnet is expending energy until it foots up an almost inconceivable total of actual power. Not alone that, but the magnet of one pound lifting power to-day may and will be stronger to-morrow.

Where does all this really tremendous amount of energy come from ? By what inscrutable process does the mere magnetization of a bar of steel make of it a machine for the transformation of energy ? Not a reactory or storage device, which, like a steel spring, honestly gives back approximately all it has received, but a perpetual transforming or converting machine. There is a hidden process going on of some kind ; energy is going into the magnet all the time it is doing work—energy in some form. Where does it come from—gravity ? atmosphere ? solar rays ? earth currents ? Who can say ? It is a great problem, worthy of a lifetime of indefatigable research. It is a microbe, and it will be discovered, and the discovery will make electricity the queen of nature's forces, and steam will become a dim vision of the dark ages of the past.—*Electrical Review.*

---

An egg is said to contain as much nourishment as a pound and one ounce of cherries, a pound and a quarter of grapes, a pound and a half of russet apples, two pounds of gooseberries, and four pounds of pears, and that 114 pounds of grapes, 127 pounds of russet apples, 192 pounds of pears, and 327 pounds of plums are equal in nourishment to 100 pounds of potatoes.

A cure for catarrh is recommended by a druggist, who pronounces it an absolute remedy. It is as follows : To an ounce of glycerine add fifteen or twenty drops of carbolic acid, and thoroughly apply with a small sponge, to be found at all drug stores, known as the ear sponge. The stimulating and antiseptic properties of the carbolic acid, combined with the soothing qualities of the glycerine, produce the most happy results. This remedy affords almost immediate relief to an ordinary cold.

### GOLDFISH HAVE FUN WITH THE TURTLE.

Washington *Critic* has the following : Fishes are not ordinarily supposed to be gifted with any great amount of intelligence, but an accident which occurred in a Washington home a few days ago proves that they have a keen sense of humor and are fond of practical joking. One of the young women of the house in question has for pets a baby mud-turtle and several goldfishes. The turtle is frequently placed in the same tank with the fish. The other day he lay floating on the surface of the water asleep and with his fore feet out.

The goldfish saw in this a good chance to play a trick on Mr. Turtle, and after putting their heads together a few minutes, they divided into four groups, and, seizing his feet in their mouths, dragged him to the botton of the tank. When awakened by his sudden immersion, he had considerable difficulty in shaking himself free from his tormentors. There cannot be the slightest doubt that the fishes had some means of communicating their ideas to each other, for it was plain to those who observed the incident that the trick was the result of preconcerted action.

---

A RECIPE for whitewash, suitable for out-buildings on a farm, something that will not wash or rub off and not injure trees, can be tinted: For one barrel of colorwash use half a bushel white lime, three pecks hydraulic cement, 10 pounds umber, 10 pounds ochre, one pound venetian red, one quarter-pound lamp black. Slake the lime, cut the lamp black with vinegar and mix well together; then add the cement and fill the barrel with water. Let it stand 12 hours before using, and stir it frequently while putting it on.

RECIPE FOR LAUNDRYING SHIRTS.—A laundress of wide experience writes that the doing of that most difficult thing of all in laundry work—the doing up shirt bosoms—may be made highly successful by observing the following procedure : Enough cold starch to last several months may be made of one ounce of white laundry wax, two ounces of borax, one teacupful of water and three teacupfuls of starch. The borax and wax are dissolved in water, sufficiently heated for the purpose, but not hot enough to scald the starch ; into this mix the pulverized starch after passing it through a flour sieve. In using, take a teaspoonful of this prepared starch and dissolve in water that is not cold enough to prevent the wax from softening. The hot starch is made, not very thick, and a teaspoonful is allowed to a shirt bosom, the hotter the liquid is the better. Apply a tablespoonful at a time, rubbing in well before putting on more, and after the right side will take up no more, apply to the under side. Unless the starch is well rubbed in, the iron will stick and specks and blisters will appear. The hot starching is done first, the bosom is allowed to dry and then the cold starching is done by dipping the bosoms in the liquid, wringing out and rubbing slightly. After an hour or so, iron, first rubbing the bosom carefully with a cloth wrung out in hot water, to equalize the starch on the surface. A thin cloth is to be laid over the bosom the first time the iron is passed over it. When this is removed, dampen tne surface of the bosom a little, and finally iron carefully until the finish is satisfactory. Let the outside cover of the ironing board be woolen cloth and the bosom will not stick to it.

### AN INDIAN'S ARGUMENT FOR VEGETARIANISM.

An address made many years ago by an Indian Chief to his people, exhorted them to take up agriculture, and presents the practical side of the subject of vegetarianism, which must have had no small weight with those to whom it was addressed: "See ye not that the pale-faces feed on grains, while we feed on flesh? that the flesh takes thirty months to grow, and that it is often scarce? that every one of those wonderful grains which they strew into the earth yields to them a thousand-fold return? that the flesh on which we live has four legs to flee from us, while we have only two to run after it? that the grains remain and grow up in the spot where the pale-face plants them? that winter, which is the season of our toilsome hunting, is to them a season of rest? No wonder, then, that they have so many children, and live longer than we do. Therefore I say to every one of you who will listen, that before the cedars of our village shall have died of age, and the maples of the valley have ceased to give us sugar, the race of the corn-eaters will have destroyed the race of the flesh-eaters, unless the hunter should resolve to exchange his wild pursuit for that of the husbandman."

---

House Cleaning.—Carpets which have become spotted by having liquids, etc., spilt upon them, may be freshened and the spots removed by going over the surface with a cloth dipped in warm water in which a little ammonia has been put. Painted or enamelled furniture is washed with ammonia water or soap and water. Polished mahogany or other polished furniture or iron bedsteads, etc., may be cleaned with beeswax and turpentine applied with a small piece of flannel, then polished by vigorous rubbing with dry dusters. The beeswax is finely shred into a small jar and turpentine poured over it, it is allowed to stand over night and will be ready for use in the morning. Thin oilcloth, such as is used for the edges of bedrooms, may be cleaned with the beeswax and turpentine too. All bedroom ware must be washed, then scalded with boiling water and dried with a clean cloth. Bedroom carpets, after being well shaken or beaten, are much improved and freshened by being drawn over grass and allowed to remain on it during the sunny part of the day, if the colors be such as will not fade readily, The walls, if not to be repapered, are rubbed down with dry dusters, and the paints washed with warm water and soap. Grained woodwork is washed with chamois leather and cold water. All the bedding is carried out of the room, and, if a sunny day, it is a capital plan to spread mattresses, bed, bolsters, and pillows out on sheets and let them lie in the sunshine till the middle of the afternoon. In some country districts it is quite a common custom during the summer for the cottagers to bring out their beds and hang them over a gate or wall where they are allowed to remain during the heat of the day. I should imagine it to be the very best way of airing and sweetening bedding. If the floor be a wooden one, it is washed with warm water and soap, and, if only part be carpeted, and the white boards show in places, it will be found a good plan to go over the whole floor again with clean cold water. This whitens the boards. In many houses it is becoming quite customary to take up the bedroom carpets entirely for the summer, and simply use a few rugs instead. The floor is then washed weekly. It has a very clean and cool effect during the heat of the summer. It is a good plan to have two sets of hangings for the bed and windows, one for winter use the other for summer. Personally, I prefer a bed without hangings of any kind except a single valance round it, and even this may be dispensed with in summer. Supposing, however, that our imaginary bed has hangings of damask or dark cretonne for winter, with window curtains to match ; a nice change would be to have one of the pretty light new chintzes for the bed hangings. The window curtains might be the simple white, plain, or spotted muslin, or any of the pretty cream or white fabrics sold for the purpose, patterns of which can be procured at any large drapers.

THE HAIR.—The hair is the covering of the roof of "the home of thought and palace of the soul." Where baldness, which sometimes occurs in quite young persons, is hereditary, it is doubtful if any thing can be done to prevent or remedy it. Avoid "restoratives" and other nostrums, and, as a rule, do not use pomatums or oils upon the head. The thorough use of a moderately stiff brush will greatly promote the health of the scalp and prevent the falling of the hair without other application. The hair should be occasionally washed, and if there is much dandruff, the yolk of an egg will be most efficient in removing it. Work the egg with the fingers well into the hair, a little at a time, to bring it in contact with the scalp ; then wash it out thoroughly with water, and the hair will be beautifully clean and soft. Avoid all shampooing liquids ; those used by barbers are strong potash solutions. They call it "Salts of Wormwood" and "Salts of Tartar," and use it without knowing its real nature. It is very effective in cleaning but ruinous to the hair. If the falling of the hair is not prevented by thorough brushing, some stimulating application may be made. Half an ounce of the tincture of cantharides added to a quart of bay rum will answer better than most "hair tonics." But the mode of dressing the hair must be controlled almost entirely by the fashion. It will be considered by many of our lady readers a necessity to dress the hair in the fashion of the moment, but we should endeavor to counteract, by careful treatment, any injurious effects, such as overheating of the scalp, which produces dandruff, irritation, and possible baldness. Whatever style is adopted during the day and evening, the hair should be given the utmost freedom during the night. All cannot employ artists to direct the efforts of the hair-dressing maid, but every one can see to it that simplicity and an appropriate ensemble are presented. Nothing is more unseemly than to see a noble, dignified face marred, and its true beauty destroyed by some coquettish or frivolous arrangement of the hair wholly out of keeping with the general bearing of the wearer.

Fortunately, the custom of the hour demands that the short-comings of one head shall be supplied by some other head, and from this necessity has grown up the present great trade in human hair. It is estimated that more than a million pounds of human hair is annually marketed, to say nothing of the product of the home market, which finds its supply largely through the periodical craze for "short hair," which American women experience, when the product of the barber's shears generally finds its way to other fields of adornment.

### WOMAN.

The well known Mrs. Livermore, in a lecture delivered some years ago, said with all due reverence, "God evidently knew what he was about when he created little boys, but when little girls came into existence they were so poorly fashioned that from their tenderest years until they laid life's weary burden down, they were considered fit subjects for improvement in form." Unfortunately, this would seem to be true, for instead of being allowed the freedom of childhood, the little girl is early taught that if she would have a good form she must wear certain articles of clothing to produce the desired result. It is this constant compression of the vital organs that is productive of such dire results ; this constant striving after artificial beauty that has made American women what they are to-day. Higher education will bring about better results; instead of unfitting our girls for the duties of motherhood, it will develope a better and truer idea of feminine beauty. It will bring into favor health and strength of limb and body, and instead of invalidism being a mark of refinement, it will be regarded as almost sinful to be ill. When mothers become learned enough to permit their daughters to grow up as God and Nature intended they should do, then will we have a better, truer class of men. Surely the cultivation of intellectual powers will have the tendency to destroy some of the fallacies of to-day. Men do not go to perform the duties of the day so begirt that to draw a long breath would be impossible. Their muscles are not cramped, and their limbs have a freedom of movement unknown to the weaker sex.

### TELL ME, MY HEART.

**Translated from the German of Friedrich Halm, for Hall's Journal of Health.**

Of thee, my heart, would I enquire,
What is love, wilt tell ?
Two souls with only one desire,
Two hearts that feed each other's fire,
And with each other dwell.

And tell me whence love comes, I pray ?
It comes, and it is here,—
And whither doth it vanish, say ?
It were not love to pass away,
For love is ever near.

And prithee, what is love that's pure ?
'Tis that which self denies.
And when is love accounted sure,
The firmest rooted to endure ?
'Tis when it stillest lies.

